# Legume consumption among Israeli adults: results from a national health and nutrition survey

**DOI:** 10.1007/s00394-026-03984-y

**Published:** 2026-05-08

**Authors:** Orit Ofir, Chen Dor, Aliza H. Stark, Rita Dichtiar, Tal Shimony, Yael Bar-Zeev, Tali Sinai

**Affiliations:** 1 The Robert H. Smith Faculty of Agriculture, Food and Environment, School of Nutritional Sciences, Institute of Biochemistry, Food Science and Nutrition, The Hebrew University of Jerusalem, Rehovot, Israel; 2 Nutrition Research Department, Israel Center for Disease Control, Israel Ministry of Health, Ramat Gan, Israel; 3 Faculty of Medicine, Braun School of Public Health and Community Medicine, The Hebrew University of Jerusalem, Jerusalem, Israel

**Keywords:** Legume consumption, Pulse consumption, Sustainable diets, Health and Nutrition National Survey, Chronic disease prevention

## Abstract

**Purpose:**

Substantial health and environmental benefits of legume consumption are reflected in dietary recommendations worldwide. However, data regarding legume intake are limited, particularly in Mediterranean countries. This study aimed to estimate and characterize legume consumption in the Israeli population.

**Methods:**

This cross-sectional study used data from the Israeli Health and Nutrition Survey (2014–2016), a nationally representative sample of the population aged 18–64 years. A personal, face-to-face interview was conducted in the interviewee’s home using a structured comprehensive questionnaire. Single 24 h dietary recalls (n = 2808) were evaluated to identify legume consumers, including quantity and type of legumes consumed. Consumers were defined as respondents who reported intake of any amount of legumes (beans, lentils, peas and soy) or legume-containing products. Demographics, health conditions, and lifestyle habits were compared between legume consumers and non-consumers. Multivariable logistic regression identified factors associated with being a legume consumer.

**Results:**

Legumes were consumed by 31.1% of respondents. Median (interquartile range) daily legume intake among consumers was 40.8g (20.4–74.0), equivalent to ~ 0.25 cup per day. Chickpeas were most commonly consumed (67.0%), followed by lentils (14.5%) and dry beans (12.2%). Legume consumers were less likely to have chronic comorbidities [OR 0.54 (95% CI 0.37-0.78)], and more likely to be male [OR 1.41 (95% CI 1.2-1.65)] and born in Israel [OR 1.24 (95% CI 1.01-1.51)].

**Conclusions:**

Legume consumption among Israeli adults was substantially below current guidelines. Further studies evaluating legume consumption worldwide and specifically in Mediterranean countries are needed, alongside public health strategies promoting legume consumption as part of healthy, sustainable dietary patterns.

## Introduction

Substantial scientific evidence supports the health benefits and environmental advantages of legume consumption [1–4]. Legumes are members of the Fabaceae family and include oilseed legumes (soybeans and peanuts) and non-oilseed legumes, the latter comprised of pulses (dry beans, peas, lentils and chickpeas) and fresh legumes (e.g., fresh beans) [5]. In a nutritional context, legumes typically refer to dry pulses and soybeans [6–8]. Legumes provide protein, B vitamins, and minerals such as iron and zinc [9]. Additionally, they are low in saturated fat and high in dietary fiber and phytochemicals—unlike animal protein sources [9]. This unique nutritional profile may account for the health benefits attributed to legume consumption in the prevention of cardiovascular diseases [10–13], type 2 diabetes [14], colorectal cancer [13] and a decrease in all-cause and stroke mortality [15]. Legume consumption is also recognized as an important factor in the current global efforts to diminish the environmental crisis by decreasing greenhouse gas emissions and reducing use of water, land, and fuel in comparison to animal protein production [1, 16, 17].

The health and environmental merits of legumes have been reflected in dietary guidelines worldwide. The EAT–Lancet Commission global recommendations for healthy and sustainable diets, emphasize legumes as the primary protein source, with daily consumption of ~ 2/3 cup (~ 130 g) of cooked pulses and 25 g of soy foods [1]. The Dietary Guidelines of the United Kingdom [18], Canada [19], New Zealand [20] and The 2025 Advisory Committee for the Dietary Guidelines for Americans 2025–2030 [21] have also declared legumes as a preferred source of protein. The 2020 Israeli Dietary Guidelines include a recommendation for daily legume intake [8].

There are limited data available regarding legume consumption around the world. Existing evidence indicates a considerable gap between recommended and actual legume intake [22–27]. Limitations of the literature include inconsistencies in reported measures and variability in the classification of foods considered as legumes. A review [22] based on the 2018 Global Dietary Database [28] found that only 11 of 93 countries had a median legume intake exceeding 50 g (~ 1/4 cup/day) in the general population. Yet, these estimates are modelled rather than derived from primary consumption data, and do not report intake among legume consumers or the proportion of consumers within the population [28]. Nationally representative surveys providing such information exist but, to the best of our knowledge, are not from Mediterranean countries [23–27]. In the United States, the National Health and Nutrition Examination Survey (NHANES 2017–2018) reported that 20.5% of adults consumed legumes, with a mean intake of ~ 0.3 cup/day (~ 65 g) among consumers [23]. Specifically, the proportion of peas and lentils consumers was 6% [29]. In Australia (2011–2012), 12.3% were identified as legume consumers, with a median intake of ~ 0.2 cup/day (~ 40 g) [24]. In contrast, substantially higher rates were observed in urban Latin American populations (2014–2015), where approximately 60% consumed pulses (mean ~ 0.5 cup/day, ~ 100 g) [25]. In Sweden (2011), 44% were identified as legume consumers (median ~ 0.25 cup/day, ~ 40 g), although the definition of legumes was broad, including peanuts and fresh legumes [26].

To better understand the gap between current dietary guidelines and actual intake, comprehensive data on legume consumption from nationally representative surveys are needed. Mediterranean countries are of particular interest, as legumes have traditionally been an integral component of the Mediterranean diet [30]. Notably, Israel's legume consumption may be perceived as sufficient, being a Mediterranean country known for its legume-based national dishes [31]. However, whether legumes are consumed in adequate amounts in Mediterranean populations remains unclear, as comprehensive nationally representative data from this region are lacking. The current study examined legume consumption among the Israeli adult population and characterized the legume consumer using the data of the most recent nationally representative Health and Nutrition Survey. The specific aims were to (1) determine the percentage of legume consumers among Israeli adults, (2) quantify legume intake among consumers and in the general population, (3) characterize legume consumption patterns by type and form, including specific chickpea and soy-based foods, and lastly, (4) compare sociodemographic and health-related characteristics between legume consumers and non-consumers. We hypothesized that legume consumption in Israel would be substantially below recommended levels and consumers would report less morbidities.

## Method

### Study design and population

This cross-sectional study was based on secondary data analysis obtained from the Second National Health and Nutrition Survey. The survey was conducted by the Ministry of Health (the Israel Center for Disease Control and the Nutrition Division), and the Central Bureau of Statistics, between March 2014 and May 2016 [32]. This was a nationally representative sample of the population aged 18–64 years and included 2,957 participants (52.9% women). The survey was approved by the Ethics Committee of the Ministry of Health and written informed consent was obtained upon enrolment. A personal, face-to-face interview was conducted in the interviewee’s home using a structured comprehensive questionnaire including socio-demographic characteristics, health conditions and lifestyle habits. The survey questionnaire underwent pretesting and construct validity assessment. Furthermore, standardized procedures and continuous monitoring during data collection and entry ensured accuracy and maintained data quality. Detailed descriptions of the survey design, procedures and questionnaires, can be found online [32]. Briefly, a random stratum sampling was executed, according to population group and locality. In addition, the data were weighted for the total population by weighting factors provided by the Central Bureau of Statistics, based upon age, sex, education, place of birth, population group, and dietary recall day. The questionnaire included a single 24 h dietary recall, wherein the interviewers asked the participants to describe their food intake on the day prior to the interview. In order to assist the participants in identifying foods, a visual guide containing numerous images of Israeli dishes was used. The interviewers also used measuring cups, tablespoons and teaspoons in order to help respondents estimate the quantities of food consumed. A total of 149 participants were excluded from this study due to missing data on dietary intake (n = 53) and outlying diet records defined as having an estimated energy intake greater than 5000 kcal (n = 14) or lower than 500 kcal (n = 82). Therefore, the final sample for this study was n = 2,808.

### Data collection

#### Participant’s characteristics

Data were self-reported and collected from survey responses, education level was dichotomized to a high school level education or less (≤ 12 years) or higher education (> 12 years). Place of birth was defined as Israel vs. Other. Total household income was dichotomized to below and above the poverty line (~ 3100 New Israeli Shekels per person) [33].Participants who performed at least 150 min of moderate-intensity physical activity or 75 min of vigorous-intensity physical activity per week, or an equivalent combination of moderate- and vigorous-intensity activity, were identified as meeting the WHO recommendations for adults [34]. Smoking status was dichotomized to never or ever smoked (current and former smoker combined). Alcohol consumption was recorded (type, frequency, and amount) and calculated as grams alcohol/wk. This was categorized to participants who consumed at least one alcoholic beverage a week, of at least 14 g of alcohol (according to the Centers for Disease Control and Prevention (CDC) definition [35]); participants who consumed less than one alcoholic beverage a week, and those who did not consume alcohol on a weekly basis. Comorbidity was defined as reporting a physician’s diagnosis of two or more of the following conditions: hypertension, diabetes, ischemic heart disease, stroke, non-alcoholic fatty liver disease (NAFLD) and cancer [36, 37]. Body mass index (BMI) was calculated from measured weight and height as "weight [kg]/ height [m^2^]”. Self-reported data were used for individuals with missing information on weight and/or height (25.4%, n = 684). BMI was assessed as a continuous variable and was also categorized according to the World Health Organization (WHO) BMI categories: underweight (BMI < 18.5 kg/m^2^), normal weight (BMI 18.5 to < 25 kg/m^2^), overweight (BMI 25 to < 30 kg/m^2^) and obesity (BMI ≥ 30 kg/m^2^) [38].

#### Legume intake

In order to calculate the total legume intake from the single 24-h dietary recall for each participant, legume-containing foods, products, and recipes were retrieved from the Israeli nutritional database, and categorized by legume type. Recipes for composite dishes were collected, with legumes listed in dry form; accepted legume-specific yield factors were applied to estimate cooked weights (e.g., a factor of 2.7 for lentils converts 100 g of dry lentils to 270 g cooked) [39]. The weight of the legume consumed was calculated based on amount eaten of the specific dish. Chickpeas and soy were further categorized according to specific products. Legume consumers were defined as respondents who reported consuming any amount of legumes (beans, lentils, peas, chickpeas and soy; excluding green peas and fresh beans and peanuts) or legume-containing products. The proportion that just consumed pulses (beans, lentils, peas, chickpeas; soy excluded) was also calculated.

#### Data analysis

The mean and standard deviation (SD) or median and interquartile range (IQR) of legume intake were determined and the percentage of legume consumers was calculated. Variables potentially influencing intake were compared between legume consumers and non-consumers using Chi-square tests for categorical variables and Mann–Whitney U tests for continuous variables. Multivariable logistic regression was used to determine which factors increased the likelihood of being a legume consumer; odds ratios (ORs) and 95% confidence intervals (95% CIs) are presented. Variables significantly associated with the dependent variable (*p* < 0.05) in the bivariate analysis were included in the multivariable logistic regression model Age (in increments of ten years) and sex were included in the model as universal confounders, along with variables that were significantly associated with legume consumption in the bivariate analysis (*p* < 0.05); specifically, place of birth and the presence of comorbidities (two or more coexisting conditions in an individual). Two-tailed tests were run, and statistical significance was set at *p* < 0.05. Statistical analysis was performed utilizing SAS statistical software, version 9.4 (SAS Institute, Cary, NC).

## Results

Among the 2808 participants included in the study (mean age 39.8 ± 12.4 years, 50.1% women), approximately half (52.4%, n = 1549) had a higher education (> 12 years) and 16.0% (n = 468) lived below the poverty line. Almost half (48.7%, n = 1297) were overweight or obese and 7.6% (n = 206) reported having two or more comorbidities. A third (33.4%, n = 911) of the participants reported meeting the recommendations for physical activity and 62.8% (n = 1709) reported they had never smoked (Table 1).

**Table 1:** Sociodemographic and health-related characteristics of legume consumers and non-consumers (n = 2808)

Variable	N total	All (n = 2808)	Legume consumers (n = 874)	Non- Legume consumers (n = 1934)	p value
Age, y (mean ± SD)	2808	39.8 ± 12.4	39.3 ± 12.2	40.1 ± 12.5	0.11
Sex, % women	2808	1467 (50.1)	407 (44.8)	1060 (52.5)	< 0.001
Education, % ≥ 12y	2755	1206 (47.6)	377 (46.8)	829 (47.9)	0.99
Income, % < poverty line	2158	468 (16.0)	150 (16.9)	318 (15.7)	0.86
Place of birth, % Israel	2808	2134 (76.6)	694 (80.9)	1440 (74.7)	0.005
BMI, kg/m^2^ (mean ± SD)	2697	25.5 ± 5	25.5 ± 5.2	25.6 ± 4.9	0.32
BMI categories^1^	2697				0.19
Underweight, %	115	115 (4.5)	45 (5.4)	70 (4.1)	
Normal weight, %	1285	1285 (46.8)	401 (47.2)	884 (46.7)	
Overweight, %	833	833 (31.2)	247 (29.1)	586 (32.1)	
Obese, %	464	464 (17.5)	152 (18.3)	312 (17.1)	
Comorbidity^2^ (% yes)	2767	206 (7.6)	40 (4.7)	166 (8.9)	0.001 >
Hypertension (% yes)	2757	424 (15.9)	111 (14.1)	313 (16.7)	0.017
Diabetes (% yes)	2774	227 (8.3)	61 (6.9)	166 (9.0)	0.14
NAFLD (% yes)	2763	151 (5.3)	36 (3.9)	115 (11.5)	0.046
Ischemic heart disease (% yes)	2765	103 (3.9)	31 (3.9)	72 (3.9)	0.82
Cancer (% yes)	2768	84 (2.8)	17 (1.7)	67 (3.4)	0.028
Stroke (% yes)	2767	46 (1.6)	7 (0.7)	39 (1.9)	0.018
Physical activity as recommended ^3^, %	2732	911 (33.4)	176 (30.1)	735 (34.1)	0.97
Smoking status, % Never smoked	2746	1709 (62.8)	535 (63.5)	1174 (62.4)	0.99
Weekly alcohol serving	2718				0.18
Non consumers, %	1229	1229 (45.7)	399 (47.9)	830 (44.8)	
< 1 portion/week, %	711	711 (26.6)	227 (26.4)	484 (26.6)	
≥ 1 portion/week, %	778	778 (27.7)	223 (25.7)	555 (28.6)	

Calculated with the application of sample weights of the Second Israeli National Health and Nutrition Survey. Data are mean ± SD or n (%)

^1^Underweight- BMI < 18.5 kg/m2; normal weight- BMI 18.5 to < 25 kg/m2; overweight- BMI 25 to < 30 kg/m2; obesity BMI ≥ 30 kg/m2

^2^Diagnosed with two or more of the following conditions: hypertension, diabetes, ischemic heart disease, stroke, non-alcoholic fatty liver disease and cancer (self-reported physician diagnosis)

^3^At least 150 min of moderate-intensity physical activity or 75 min of vigorous-intensity physical activity per week, or an equivalent combination of moderate- and vigorous-intensity activity, was identified as meeting the WHO recommendations for adults

*BMI* Body Mass Index; *NAFLD* Non-alcoholic fatty liver disease

Legumes were consumed by 31.1% (n = 874) of the participants, and pulses were consumed by 28.4% (n = 791) of participants. Median (IQR) legume intake among legume consumers was 40.8 g (20.4, 74.6). Median (IQR) legume intake among the general population (legume and non-legume consumers) was 0 g (0, 19.5). The most frequently consumed legume was chickpea (68.1% of legume consumers), followed by lentils (13.5%) and dry beans (12.7%). The least frequently consumed legumes were mung beans and lupine (0.2%; 0.1%, respectively). The legumes consumed in the highest amounts were lupine with a median (IQR) daily intake of 60 g (60.0, 75.0), lentils: 54.7 g (19.5, 111.4), beans: 50 g (33.3, 97.1), and fava beans: 50 g (29.17, 89.77) (Table 2). Hummus was the most commonly consumed form of legumes, reported by over half (55.2%) of legume consumers and by 81.1% of chickpea consumers, however in relatively small amounts [27.2 g (20.4- 49.8)] (Table 3). Soy was eaten by 8.8% of legume consumers and soy drinks were the most frequently used product, drunk by 58.0% of those reporting soy intake (Table 3).

**Table 2:** Characteristics of consumption by legume variety among legume consumers (n = 874)

Legume variety	Consumers n (%)	Consumption (g/day) median (IQR)
Chickpeas	586 (67.0)	34.0 (20.4, 62.0)
Lentils	127 (14.5)	54.7 (19.5, 111.4)
Beans	107 (12.2)	50.0 (33.3, 97.1)
Soy	83 (9.5)	50.0 (29.2, 89.8)
Dry peas	33 (3.8)	36.7 (23.9, 50.0)
Fava bean	12 (1.4)	50.0 (29.2, 89.8)
Mung beans	2 (0.2)	39.0 (13.4, 39.0)
Lupine	2 (0.2)	60.0 (60.0, 75.0)

IQR- interquartile range (Q_1_, Q_3_)

**Table 3:** Characteristics of consumption by Chickpea (n = 586) and Soy (n = 83) variety

Legume variety	Consumers (%)	Consumption (g/day) median (IQR)
Chickpea variety (n = 586)		
Hummus	462 (81.1)	27.2 (20.4, 49.8)
Chickpea beans	100 (15.0)	21.0 (8.8, 57.3)
Falafel	74 (13.5)	59.4 (38.8, 77.6)
Chickpea flour	5 (0.7)	32.4 (19.0, 36.0)
Roasted chickpea	1 (0.1)	124.0 (124.0, 124.0)
Soy variety (n = 83)		
Soy milk	49 (58.0)	15.6 (7.8, 23.4)
Tofu	16 (19.5)	32.7 (30.0, 66.3)
Edamame	6 (9.3)	120.0 (18.0, 350.0)
Soy yogurt	6 (5.6)	16.6 (19.8, 47.5)
Miso	4 (4.9)	250.0 (50.0, 356.0)
Soy chunks	2 (2.4)	146.0 (53.5, 146.0)
Soy flour	2 (3.0)	44.2 (5.2, 44.2)
Soybeans	1 (1.6)	5.0 (5.0, 5.0)

IQR- interquartile range (Q_1_, Q_3_)

In the bivariate analysis, compared to non-legume consumers, legume consumers were more likely to be men (47.5% vs. 55.2%, *p* < 0.001) and to be born in Israel (74.6% vs. 80.8%, p = 0.005). Both groups were similar in age, level of education and income. Legume consumers were less likely to report a physician’s diagnosis of hypertension (14.1% vs. 16.7%, p = 0.017), NAFLD (3.9% vs. 11.5%, p = 0.046), stroke (0.7% vs. 1.9%, p = 0.018), cancer (1.7% vs. 3.4%, p = 0.028), and overall comorbidity (4.7% vs. 8.9%, *p* 0.001 >). BMI, smoking status, alcohol consumption, and adherence to physical activity recommendations did not differ between the groups (Table 1).

**Fig. 1:**
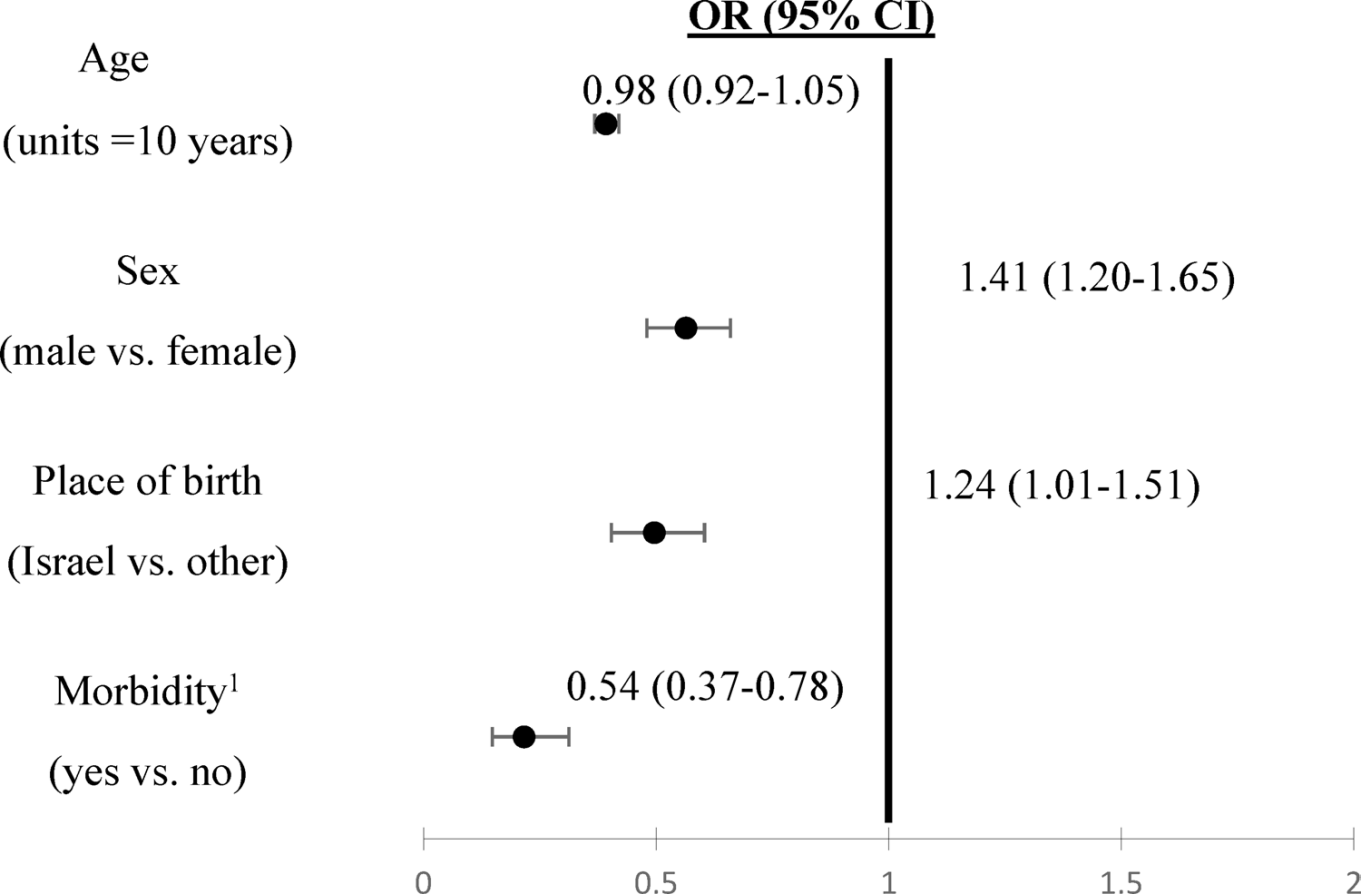
Factors associated with legume consumption among Israeli adults. ^1^ Diagnosed with two or more of the following conditions: hypertension, diabetes, ischemic heart disease, stroke, non-alcoholic fatty liver disease, and cancer (self-reported physician diagnosis)

Figure 1 presents the multivariable logistic regression results for factors associated with being a legume consumer. Legume consumption was associated with 46% lower odds to suffer from comorbidities [OR 0.54 (95% CI 0.37-0.78), p = 0.001], 41% higher odds to be male [(OR 1.41 (95% CI 1.2-1.65), p = < 0.001)], and 24% higher odds to be born in Israel [(OR 1.24 (95% CI 1.01-1.51) p = 0.038)].

## Discussion

This nationally representative sample of Israeli adults indicates that legume consumption in Israel is substantially below the current EAT–Lancet Commission global recommendations of ~ 2/3 of a cup per day [1]. Less than a third of the participants reported eating legumes with a median intake of approximately 0.25 cup/day. In addition, more than half of legume consumers reported consuming legumes in the form of hummus (Chickpea spread), with an average intake of one tablespoon per day. Legume consumers were less likely to report comorbidities and more likely to be male and born in Israel.

Despite the low proportion of legume consumers in this study, it was higher than the rates previously reported in Australia [24], USA [23] and Canada [27] but lower than Latin America [25]. It is important to note that legume classification differed between studies, thus limiting comparison. Legumes were defined in this study as pulses (beans, lentils, peas) and soy, similarly to the Australian survey in which the rate of legume consumers was considerably lower (12.3%) [24]. The US [23] and Canadian [27] surveys analyzed solely pulse consumption, reporting 20.5% and 13% consumers, respectively. These rates were lower than the 28.4% pulses consumption rate found in this study. Conversely, an almost twofold higher rate of 60% legume consumers was found among Latin Americans [25] with legumes defined as beans, lentils, chickpeas and green peas. A rate of 44% legume consumers was reported in Sweden [26]; however, the legume definition in this study was exceptionally broad and included foods typically classified as vegetables (i.e., fresh beans, snap peas and sprouts) or as nuts (i.e., peanuts and peanut butter). Additionally, the Swedish study recorded a longer dietary intake period of 4 days, which further limits direct comparison with our findings.

Among legume consumers, the low legume intake of ~ 0.25 cup/day observed in this study is comparable to that reported in Australia [24], USA [23] and Sweden [26]. A higher daily intake of ~ 0.5 cup and 2/3 cup was reported in Latin America [25] and Canada [27], respectively. The most frequently consumed legumes in this study were chickpeas, followed by lentils and beans, which were consumed in significantly smaller amounts. Most studies did not report the type of legume consumed, but available data show markedly different preference patterns. Beans (pinto, black and kidney) predominated in the American data, followed by lima bean, chickpeas, great northern beans and lentils, as reported by grocery store purchases by cost [23]. Mung beans were the most frequently consumed pulse in Canada, followed by kidney beans, lentils and chickpeas [27].

Legume consumption in Israel and other high-income countries may have increased over the past decade, driven by public awareness of health, environmental, and ethical aspects of food choices and by dietary guidelines (e.g., 2020 Israeli guidelines, 2019 EAT-Lancet) [1, 8]. In particular, increased awareness of the environmental impacts of animal-based foods may have encouraged a shift toward plant-based proteins. This may have raised intake of dry or minimally processed legumes (e.g., tofu) [40], but meat substitute consumption, often based on soy or pea protein isolates, has also risen [41, 42]. These products are classified as ultra-processed foods and are therefore not aligned with dietary recommendations [1, 8]. Paradoxically, beef consumption has increased by ~ 50% since 2015, with Israel ranking fourth globally in 2021, averaging 19.6 kg beef per capita [43]. Furthermore, Israeli dietitians seldomly meet national dietary recommendations for legume intake, with less than a third advising most of their patients to consume legumes daily and only ~ 5% doing so themselves [44, 45], suggesting that current Israeli legume intake likely remains well below guidelines. Lastly, it is important to note that demographic changes and considerable immigration to Israel, together with marked dietary diversity across population groups could further affect legume consumption patterns.

In this study legume consumers were similar to non-consumers in age, level of education and income. While legume consumption was not associated with income in previous studies [23–25, 27], some studies found a positive association with education [23, 26] and age [26, 27]. In accordance with our study, BMI was not found to be associated with legume consumption in Sweden [26] and Australia [24]. Smoking, alcohol consumption and physical activity were also not associated with legume consumption in this study. To the best of our knowledge, previous studies did not report these health behaviors [23–25, 27], an exception was observed in a Swedish study, which reported an inverse association between legume and alcohol consumption [26]. The lack of association with education and health related behaviors observed in Israel might be related to the overall unsatisfactory consumption patterns among legume consumers. It may also reflect a lower public awareness regarding the health benefits of legumes compared to other food groups, such as vegetables, whose intake is typically associated with higher education and health-related behaviors [46, 47].

Legume consumers in this study were less likely to report having two or more morbidities. Non-communicable diseases were not reported in other studies [23–27]; however, substantial evidence from cohort and clinical research has demonstrated the role of legume consumption in the prevention and management of chronic diseases [10, 11, 13, 14]. None-the-less, as this is a cross-sectional study, these data cannot determine causality.

Legume consumers in this study were more likely to be men and to be born in Israel. Similarly, more men were found among Latin American legume consumers [25]; however, no association to sex was found in other studies [23, 24, 26, 27]. The Israeli cuisine traditionally incorporates legume-based dishes [31], while most of the participants who were not born in Israel reported being born in Europe, where legumes are less common in the cuisine [48]. Ethnicity was found to be associated with legume consumption also among Hispanic individuals in USA [23] and among individuals of Asian, Arabic and Latin descent in Canada [27]. Key barriers to legume consumption include a long preparation time [49, 50], concerns about flatulence [49, 51] and insufficient knowledge on preparation methods [48, 50, 52, 53].

The results reported here show a significant association between legume consumption and lower morbidity. This supports the critical role legumes play in human health. Along with the positive impact on environmental sustainability, developing programs to increase legume consumption is timely and essential. Interventions targeting health professionals as “agents of change” may achieve broader and more scalable impact than those aimed solely at the general public [54]. Dietitians are a particularly relevant target group given their influence on consumption patterns across clinical, institutional, food industry, public health, policy, and academic settings [55, 56]. A recent randomized controlled trial [44] found that an online training program improved Israeli dietitians’ legume counseling practices, attitudes, knowledge and personal intake. Increasing legume consumption should also be supported by media campaigns and policies implementing dietary guidelines in institutional food settings.

The strengths of this study include the use of data from a large nationally representative sample. Internal validity was strengthened by the use of validated measurement tools, standardized protocols, and interviews conducted by trained personnel. To the best of our knowledge, this is the first study executed in a Mediterranean country to comprehensively estimate and characterize legume consumption, providing important data regarding the current implementation of this aspect of the Mediterranean diet. The study has some limitations; despite it being a well-known and validated dietary assessment method, a single 24 h dietary recall may not reflect the habitual diet. The large sample size and the data representing different days across the week aid in mitigating this limitation. Secondly, although this study is based upon the most recent national survey performed among Israeli adults, the data were collected between 2014 and 2016. However, currently available data from other countries are similarly from these years [23, 25] or earlier [24, 26, 27]. While awareness of the advantages of legumes and availability of minimally processed legume products have increased over the past decade, current consumption in Israel likely remains substantially below guidelines.

## Conclusions

This study is the first to estimate and characterize legume consumption in a Mediterranean country. Legume consumption among Israeli adults was substantially below current guidelines. Less than a third of participants reported eating legumes, with a median intake of approximately 0.25 cup/day. Legume consumers were more likely to be male and born in Israel, and also reported fewer comorbidities. Evaluating current consumption is a fundamental step in promoting legumes as a healthy and sustainable protein source, helping reduce the societal and economic burden of diet-related chronic diseases while supporting sustainable food systems. Further studies are needed worldwide, and particularly in Mediterranean countries, to comprehensively evaluate and routinely monitor legume intake, ideally with a standardized legume classification reflecting nutritional rather than botanical definitions. In light of these findings, interventions should be designed to increase legume intake through clinical practice, nutrition education and policy measures. Appropriate evaluation of such interventions will help to establish global evidence-based guidance.

## Data Availability

Data are available upon reasonable request from the corresponding author, and will require authorization from the Israel Ministry of Health.
